# Preparation of Monoclonal Antibodies against the Capsid Protein and Development of an Epitope-Blocking Enzyme-Linked Immunosorbent Assay for Detection of the Antibody against Porcine Circovirus 3

**DOI:** 10.3390/ani14020235

**Published:** 2024-01-11

**Authors:** Junli Wang, Baishi Lei, Wuchao Zhang, Lijie Li, Jiashuang Ji, Mandi Liu, Kuan Zhao, Wanzhe Yuan

**Affiliations:** 1 College of Veterinary Medicine, Hebei Agricultural University, Baoding 071000, China; 2 Hebei Veterinary Biotechnology Innovation Center, Hebei Agricultural University, Baoding 071000, China; 3 North China Research Center of Animal Epidemic Pathogen Biology, China Agriculture Ministry, Baoding 071000, China

**Keywords:** porcine circovirus 3, cap protein, monoclonal antibodies, B cell epitope, epitope-blocking ELISA

## Abstract

**Simple Summary:**

Research on the Cap protein is highly significant in the diagnosis, prevention, and control of porcine circovirus 3. In this study, we identified a novel B cell epitope of Cap protein using monoclonal antibody, and an epitope-blocking enzyme-linked immunosorbent assay was successfully developed to detect PCV3 antibodies in porcine sera. This established EB-ELISA has the advantages of being rapid, highly sensitive, re-producible, specific, and did not react with other porcine virus sera, which has great potential for the detection of PCV3 antiserum in porcine farms.

**Abstract:**

Porcine circovirus type 3 (PCV3) is endemic in swine worldwide and causes reproductive disorders, dermatitis and nephrotic syndrome, and multi-organ inflammation. Currently, there is a growing need for rapid and accurate diagnostic methods in disease monitoring. In this study, four monoclonal antibodies (mAbs) against PCV3 capsid proteins were prepared (mAbs 2F6, 2G8, 6E2, and 7E3). MAb 7E3, which had the highest binding affinity for the Cap protein, was chosen for further investigation. A novel B cell epitope ^110^DLDGAW^115^ was identified using mAb 7E3. An epitope-blocking (EB) enzyme-linked immunosorbent assay (ELISA) was successfully developed using horseradish-peroxidase-labeled mAb 7E3 to detect PCV3 antibodies in porcine sera. Moreover, the EB-ELISA showed no specific reaction with other porcine disease sera, and the cut-off value was defined as 35%. Compared with the commercial ELISA, the percentage agreement was 95.59%. Overall, we have developed a novel EB-ELISA method that accurately and conveniently detects PCV3 in serum, making it a valuable tool for the clinical detection of PCV3 infection.

## 1. Introduction

Porcine circovirus (PCV) belongs to the Circoviridae family and has a circular, single-stranded DNA genome. It is the smallest known virus that is able to replicate independently in mammalian cells [1]. There are four strains of PCV, named as follows: PCV1, PCV2, PCV3, and PCV4. PCV1 was first detected in the porcine kidney cell line PK-15, and its pathogenicity has not been investigated so far [1,2,3,4], which was followed by a report on PCV2 in 1998 [5]. PCV2 is pathogenic and was isolated from swine with a disease called post-weaning multisystemic wasting syndrome (PMWS) [5,6,7]. The first isolation and characterization of the PCV3 virus strain was obtained in the USA from a case of porcine dermatitis and nephropathy syndrome (PDNS) in 2016 [8], and most recently in 2019, PCV4 was first identified in swine with clinical signs similar to PDNS as a new member of PCV in China [9]. The pathogenesis of PCV3 is not well defined; however, viral DNA is frequently detected in swine, displaying various clinical conditions, such as dermatitis, nephropathy syndrome, congenital tremors, and reproductive failure [10,11,12,13,14]. Moreover, there is little direct correlation between PCV3 infection and age, as the virus has been detected in individuals of various age groups. Additionally, the duration of infection has been observed to range from 4 to 23 weeks [15].

It was discovered that the majority of PCV3 isolates were classified into three clades based on two amino acid mutations (A24V and R27K) in the Cap protein and their evolutionary relationship: PCV3a, PCV3b, and PCV3c [16,17]. PCV3 contains an ambisense, single-stranded, closed-circular DNA genome of 2000 bp [8,12,16]. Genomic DNA of PCV3 consists of three open reading frames (ORFs): ORF1, ORF2, and ORF3. ORF1 encodes a replication-related protein (Rep), the major capsid protein (Cap) of the virus was encoded by ORF2, and ORF3 encodes a protein whose function is currently unstudied [8]. The PCV3 Cap protein, composed of 214 amino acids (aa), is the key structural protein of the virus, which is only 26–36% identical to the Cap protein of PCV2 [8,10]. Cap protein is necessary for virion packaging and has been shown to be the major target of the host immune response [18,19,20]. It has been reported that purified PCV3 Cap protein has the ability to self-assemble into 10 nm virus-like particles (VLPs), which have been used as coating antigens in an indirect enzyme-linked immunosorbent assay (ELISA) [21,22]. In addition, the PCV3 Cap protein inhibits type I interferon (IFN) signaling by interacting with the host protein, which indicates that the PCV3 Cap protein can cause immunosuppression [23,24]. Several studies have reported that Cap is one of the most important PCV3 proteins and has been used as a target for detecting PCV3 infection in serological assays [8,21,25].

As there is no effective commercial vaccine to prevent and control PCV3 infection, the detection of PCV3 antibodies may indicate that the host is currently or has been infected with PCV3 in the past. Several diagnostic techniques are available for the early detection of PCV3 infection, including in situ hybridization (ISH), immunohistochemistry (IHC), PCR, and quantitative fluorescence PCR (qPCR) [8,10,18,21,26]. Among these, qPCR has been widely used to detect PCV3 DNA [27,28]. The serological diagnosis of PCV3 can be used as a complementary condition to the qPCR assay; therefore, many indirect ELISA methods based on recombinant PCV3 Cap protein have been established [8,21,25]. However, indirect ELISA also has some drawbacks. For example, it requires a highly pure antigen or secondary antibodies, making the development of direct ELISA commercial diagnostic kits more complex. Among the various immunoassays, the mAb-based blocking ELISA is highly specific for antimicrobial detection and reduces the detection rate of false positive samples when monitoring negative samples. Currently, there is no blocking ELISA for PCV3 antibody detection; accordingly, the development of a blocking ELISA using mAb is of great significance for the clinical detection of PCV3.

In the present study, monoclonal antibodies (mAbs) against *E. coli* expressing recombinant Cap protein of PCV3 were generated, and a novel B cell epitope of PCV3 was identified. The established EB-ELISA based on mAb 7E3 showed favorable specificity, high sensitivity, and a high degree of compliance with a commercial ELISA kit. This novel EB-ELISA is a rapid, simple, and cost-effective approach for the detection of PCV3 infections in pig herds.

## 2. Materials and Methods

### 2.1. Cells, Vectors, and Animals

Human embryonic kidney 293T (HEK293T) cells were maintained using 10% fetal bovine serum (FBS; Gibco, Carlsbad, CA, USA) in Dulbecco’s modified Eagle’s medium (DMEM; Gibco, Carlsbad, CA, USA). SP2/0 myeloma cells were maintained using 10% FBS in Roswell Park Memorial Institute 1640 (RPMI-1640, Solarbio, Beijing, China). The cells were all maintained in a CO_2_ incubator at 37 °C with 5% humidity. *E. coli* DH5ɑ and BL21 competent cells were acquired from Biomed (Beijing, China). The expression vectors pGEX-4T-1, pET-32a, and pCAGGS were stored in our laboratory. SPF Biotechnology Co. (Beijing, China) supplied eight 6-to 8-week-old female BALB/c mice.

### 2.2. Serum Samples

In total, 120 specific pathogen-free (SPF) swine serum samples were employed to determine the EB-ELISA cut-off value. Sixty-eight serum samples with an unknown background were used to compare the compliance rate between EB-ELISA and commercial ELISA kits (Shanghai Enzyme-Linked Biotechnology Co., Ltd., Shanghai, China). Positive sera against porcine reproductive and respiratory syndrome virus (PRRSV, *n* = 4), porcine pseudorabies virus (PRV, *n* = 4), porcine circovirus 2 (PCV2, *n* = 7), classical swine fever virus (CSFV, *n* = 4), and porcine epidemic diarrhea virus (PEDV, *n* = 5) were detected serum samples retained in our laboratory. A total of 132 serum samples derived from immunized porcine were collected from large-scale farms in Heibei province.

### 2.3. Expression of PCV3 Cap Protein

The complete coding sequence of the PCV3 Cap protein (GenBank accession No. MK580468) was synthesized and optimized (Sangon Biotech, Shanghai, China) into pGEX-4T-1 and pET-32a vectors, and the recombinant plasmids pGEX-4T-1-Cap and pET-32a-Cap were thus obtained, respectively. The expression of GST-fused Cap and 6×His-tagged Cap proteins (namely GST-Cap and His-Cap, respectively) was achieved by transforming into *E. coli* BL21. Protein expression was induced with 1 mM isopropyl-β-galactopyranoside for 6 h at 37 °C. The bacterial precipitate was collected by centrifugal force at 5000× *g* and diluted in PBS. The target proteins were lysed via ultrasonication and collected via centrifugation. The recombinant proteins in the supernatant after sonication were purified using the GST-tag and His-tag Protein Purification Kits (CoWin, Taizhou, China) following the steps outlined in the instructions. The proteins were characterized using SDS-PAGE in 12.5% polyacrylamide gels. The determination of protein concentration was conducted via a BCA protein detection kit (Solarbio, Beijing, China).

In addition, to characterize the ability of mAbs to bind to the PCV3 Cap protein in multiple ways, the PCV3 Cap protein gene was synthesized and subcloned into the pCAGGS vector (Sangon Biotech, Shanghai, China), and the resultant protein was named pCAGGS-Cap.

### 2.4. Preparation of mAbs against PCV3 Cap Protein

Complete/incomplete Freund’s adjuvant (Sigma Aldrich, Shanghai, China) equal to the amount of purified His-Cap protein (100 μg/mouse) was added, fully emulsified, and subcutaneously inoculated into mice. The mice that were primed received two additional booster doses spaced two weeks apart. Serum samples were collected from immunized mice and an indirect ELISA was performed to determine the titer of antibodies specific to Cap protein. After three immunizations, a mouse with the highest antibody potency was selected for another immunization. The mice were then humanely executed, their splenocytes were fused with SP2/0 cells, and the treated cells were cultured in RPMI 1640 medium with HAT or HT for screening. Culture supernatants from individual hybridoma clones were detected using an indirect ELISA which used the differently labeled GST-Cap protein as an encapsulated antigen. Positive hybridomas were subcloned three times using limiting dilution. The resulting stable hybridomas were then inoculated into 10-week-old female BALB/c mice to prepare the ascites fluid. Ascites fluid was purified by protein G agarose (BioRad, Beijing, China) following the steps of the manufacturer’s instructions. Identification of heavy and light chains of mAbs was achieved using the mAb isotyping ELISA kit (Biodragon, Suzhou, China). 

### 2.5. Indirect ELISA Method

For indirect ELISA, the ELISA plates were overnight-coated with purified GST-Cap proteins at 4 °C. PBS containing 0.1% Tween-20 detergent (PBST) with 5% FBS was employed to inhibit the non-specific binding of other substances at 37 °C for 1 h. The plates were washed five times with PBST and incubated with the detection antibodies for 1 h at 37 °C. After the second rinse, HRP-labeled goat anti-mouse IgG antibody (Solarbio, Beijing, China) was reacted as a secondary antibody for 1 h also at 37 °C. Tetramethylbenzidine (TMB) solution (Solarbio, Beijing, China) was used as a substrate for the color development reaction after another repeated wash. The plates were operated for 10 min at room temperature and protected from light. And color development was stopped with 2 M H_2_SO_4_. Finally, the optical density (OD) was measured at 450 nm using a spectrophotometer.

### 2.6. Western Blot

The reactivity of the antibody preparations with recombinant proteins was determined using Western blotting. Purified recombinant GST-tagged PCV3 proteins mixed with 4× loading buffer (Solarbio, Beijing, China) were subjected to SDS-PAGE electrophoresis on 12.5% polyacrylamide gels and transferred to polyvinylidene difluoride (PVDF) membranes. The membranes were incubated for 1 h at room temperature with 5% skimmed milk diluted in Tris-buffered saline containing 0.05% Tween-20 (TBST) and incubated with culture fluids of purified ascites fluid (1:2000 dilution) preparations at 4 °C overnight. After washing thrice in TBST, the membrane was co-incubated with the 1:5000 dilution of HRP-conjugated goat anti-mouse IgG antibody for 1 h at room temperature. Following three washes, PVDF membranes were exposed using chemiluminescence.

### 2.7. Immunofluorescence Assay

HEK 293T cells were grown in monolayers in a 12-well plate and transfected with 2 μg of pCAGGS-Cap plasmid using the Lipofectamine^®^ 2000 (Invitrogen, Life Technologies, Carlsbad, CA, USA) and were harvested at 72 h post-transfection. Transfected cells were washed three times with PBS and maintained with 4% formaldehyde for 40 min, and then the cells were permeabilized with 0.5% Triton X-100 for 40 min. They were washed once more, and then the cells were enclosed in PBS containing 5% BSA for 30 min and inoculated with a 1:200 dilution of the mAbs for 1 h. Subsequently, after rinsing with PBS, the cells were reacted with fluorescein isothiocyanate-conjugated goat anti-mouse IgG at a dilution of 1:200 for 1 h in the dark. Nuclei were stained with 4′, 6′-giamidino-2 phenyl-indole (Solarbio, Beijing, China) for 5 min. After the final wash, the fluorescent cells were examined under a fluorescence microscope (ZEISS, Jena, Germany).

### 2.8. Dot Blot Analysis

Approximately 2 μg of the protein or peptides was spotted onto the PVDF membrane, and the recombinant Cap protein we prepared was used as the positive control and PBS was used as the negative control. The membrane was enclosed with 5% skimmed milk in TBST for 30 min at 37 °C. Subsequently, it was incubated with mAb (1:200) in 5% skimmed milk in TBST for 1 h at 37 °C. Next, it was incubated with HRP-conjugated goat anti-mouse IgG antibody for 1 h at 37 °C. Lastly, the membrane was examined with ECL chemiluminescence solution.

### 2.9. Identification of the Linear B Cell Epitopes on Recombinant PCV3 Cap Protein

Epitopes were identified with mAbs using an indirect ELISA and dot blot assays. The PCV3 Cap gene was truncated into a series of fragments and constructed recombinant plasmids using pGEX-4T-1 as the vector. The primer information is displayed in Table S1. All the truncated GST-fused fragments were expressed and identified via Western blotting using anti-GST antibody and subjected to an indirect ELISA and dot blot method facilitated by the prepared mAb. The empty vector was used as the negative control. According to these validation results, the selected positive peptide was truncated again and identified using the same method. To validate a more precise epitope, six overlapping short peptides were artificially synthesized (Synpeptide, Nanjing, China) and analyzed using an indirect ELISA and dot blot method.

### 2.10. Development of MAb-Based EB-ELISA

The purified PCV3 Cap mAb was labeled with HRP to develop an EB-ELISA for the detection of clinical PCV3 antibodies following these steps. The plates were coated with purified GST-Cap protein and incubated at 4 °C overnight. After blocking the plates with PBST containing 5% FBS, we mixed mAb-HRP with PCV3-seropositive or PCV3-seronegative 1:1 for 100 μL and incubated the plates for 1 h at 37 °C. After the plates were washed thrice, TMB (Solarbio, Beijing, China) was added for color reaction at 37 °C for 15 min. The solution that terminates the reaction was 2 mol/L H_2_SO_4_. Finally, the plates were read at 450 nm, and the percent inhibition (PI value) of the detected samples was computed according to the formula. PI (%) = [1 − (OD_450_ value of testing serum samples/OD_450_ value of negative serum samples)] × 100%].

To optimize the testing conditions, the optimal concentration of GST-Cap protein and dilution ratio of purified antibody mAb-HRP was first identified using the checkerboard method which mixed a serial dilution of GST-Cap protein (2, 4, 8, 16, and 32 μg/mL) and purified antibody mAb-HRP (1:50, 1:100, 1:200, 1:400, and 1:800). Optimal conditions were filtered when the OD_450_ value was closest to 1.0. The dilutions of the detected porcine serum samples were optimized. Porcine serum was diluted 1:2, 1:4, 1:8, 1:16, and 1:32 for EB-ELISA detection. Finally, the blocking time of the tested porcine sera and mAb-HRP (30, 60, and 90 min) and the colorimetric reaction times (5, 10, and 15 min) were superior. The smaller the ratio of OD_450_ values between positive and negative sera (P/N), the better the results of condition optimization. The developed EB-ELISA was validated after the conditions were optimized.

### 2.11. Determination of Cut-Off Value, Specificity, Sensitivity, and Repeatability 

The cut-off value for the EB-ELISA was determined from a total of 120 negative serum samples. The value was the mean blocking rate of the 120 samples plus three standard deviations (SDs), which provided a greater likelihood of ruling out false positives.

The characterization of non-specific reactions of developed EB-ELISA methods with other positive sera against the porcine virus, PRRSV, PRV, PCV2, CSFV, and PEDV was tested to evaluate the specificity of the assay.

As for the determination of the EB-ELISA sensitivity, 132 positive porcine sera for anti-PCV3 antibodies were tested using the developed EB-ELISA. Furthermore, doubling dilutions (from 1:2 to 1:128) of six porcine serum samples positive for anti-PCV3 antibodies were tested using EB-ELISA to determine the minimum inspection limit.

Evaluation of the reproducibility of the EB-ELISA was identified using three positive samples and three negative samples. The coefficient of variance (CV) between batches was obtained by assaying the samples on three different batches of plates, and the CV within a batch was obtained by testing three replicate samples from the same batch. The ratio of the SD to the mean OD_450_ value of each group of samples is the CV value.

### 2.12. Comparisons of the EB-ELISA with the Commercial ELISA Kit

In the assessment of the conformance of the EB-ELISA established in this study with the results of commercial ELISA kits, 68 serum samples of unknown background were co-tested using two methods. The rate of overlap between the two kits was calculated by Microsoft Excel’s CORREL function.

### 2.13. Statistical Analysis

Statistical analysis and drawing were performed using GraphPad Prism software (version 9.0; GraphPad Software, Inc; San Diego, CA, USA). The kappa values were calculated by SPSS software (version 28.0), which facilitated a better determination of the correlation between the established EB-ELISA and commercial ELISA kits. 

## 3. Results

### 3.1. Expression and Purification of Recombinant PCV3 Cap Protein

**Figure 1:**
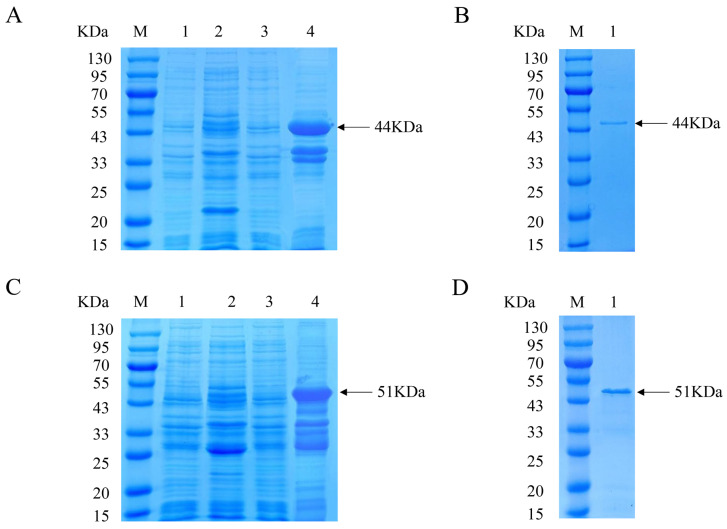
Expression and purification of recombinant PCV3 Cap protein. (**A**) SDS-PAGE analyzed the expression of His-Cap protein. Lane M, protein marker. Lane 1, uninduced *E. coli* BL21 with empty vector pET-32a. Lane 2, IPTG-induced *E. coli* BL21 with empty vector pET-32a. Lane 3, uninduced *E. coli* BL21 with pET-32a-Cap. Lane 4, IPTG-induced *E. coli* BL21 with pET-32a-Cap. (**B**) SDS-PAGE identified the purification of His-Cap protein. Lane M, protein marker. Lane 1, purified Cap protein. (**C**) SDS-PAGE identified the expression of GST-Cap protein. Lane M, protein marker. Lane 1, uninduced *E. coli* BL21 with empty vector pGEX-4T-1. Lane 2, IPTG-induced *E. coli* BL21 with empty vector pGEX-4T-1. Lane 3, uninduced *E. coli* BL21 with pGEX-4T-1-Cap. Lane 4, IPTG-induced *E. coli* BL21 with pGEX-4T-1-Cap. (**D**) SDS-PAGE identified the purification of GST-Cap protein. Lane M, protein marker. Lane 1, purified Cap protein.

The recombinant Cap protein with His and GST tags was constructed for immunization and analysis, respectively, to exclude the effect of tagged antibody production in immunized mice. The expression of the His-Cap protein was characterized via SDS-PAGE, which showed a distinct band with a molecular weight of approximately 44 kDa (Figure 1A). After Ni-chelating affinity chromatography, the purified protein was detected as a single band (Figure 1B). The prepared GST-Cap protein with an approximate molecular mass of 51 kDa was identified and purified (Figure 1C,D). The approximate size of the expressed protein was the same as expected.

### 3.2. Development of mAbs

**Figure 2:**
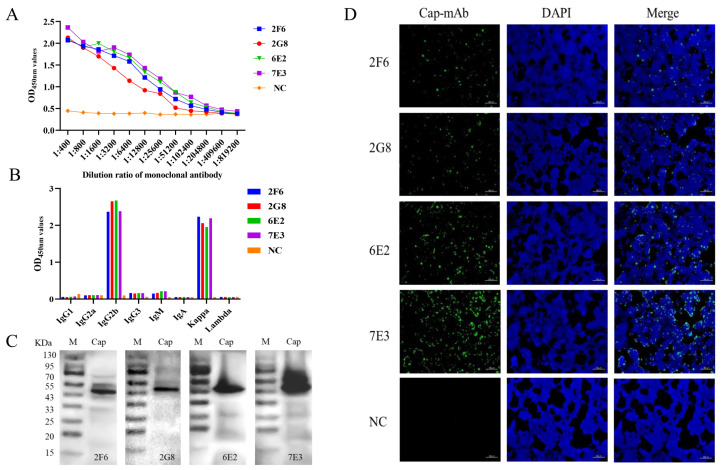
Characterization of mAbs. (**A**) Titers of four mAbs, 2F6, 2G8, 6E2, and 7E3, were 1:2.56 × 10^4^, 1:2.56 × 10^4^, 1:5.12 × 10^4^, and 1:1.024 × 10^5^, respectively. (**B**) Isotyping MAbs were characterized following the kit’s instructions. The results were read at 450 nm. (**C**) Western blot analysis of four mAbs. The four mAbs specifically reacted with recombinant GST-Cap protein at 51 kDa. (**D**) IFA analyzed mAbs affinity for recombinant Cap protein in 293T cells. The green color represents anti-Cap protein mAbs; the blue color represents nuclei. The uninoculated cells were used as negative control (NC).

BALB/c mice were immunized with purified His-Cap. The mice with the highest antibody titers were then euthanized for hybridoma production. Four positive clones specific for the GST-Cap protein, designated as 2F6, 2G8, 6E2, and 7E3, were obtained and subcloned four times by limiting dilution. Titers of the four mAbs were analyzed using an indirect ELISA (Figure 2A). The results indicated that four mAbs reacted well with the recombinant proteins specifically with high antibody potencies ranging from 1:2.56 × 10^4^ to 1:1.024 × 10^5^, and mAb 7E3 had higher potency with the Cap protein than the other mAbs. MAbs isotypes were detected using a Mouse Ig Isotyping Kit. Samples 2F6, 2G8, 6E2, and 7E3 were IgG2b with a kappa light chain (Figure 2B). These four mAbs were further verified via Western blotting (Figure 2C) and reacted strongly with the recombinant GST-Cap protein. In addition, the four mAbs showed strong reactivity with 293T cells infected with pCAGGS-Cap in the immunofluorescence assay (IFA) images (Figure 2D).

### 3.3. Epitope Mapping of PCV3 Cap Protein

**Figure 3:**
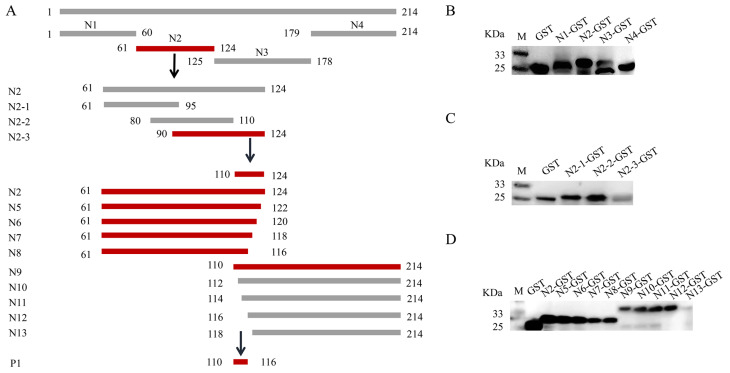
Truncation and detection of PCV3 Cap protein. (**A**) Schematic diagram of the epitope screening of PCV3 Cap protein. The Cap protein was truncated into 16 different fragments for expression of GST fusion peptides. Highlight in red indicates positive fragment in the results; highlight in gray indicates negative fragment in the results. (**B**) The truncated Cap protein N1-N4 fragments reactivity with anti-GST tag antibody analyzed by Western blot. (**C**) The truncated Cap protein N2-1-N2-3 fragments reactivity with anti-GST tag antibody analyzed by Western blot. (**D**) The truncated Cap protein N5-N13 fragments reactivity with anti-GST tag antibody identified by Western blot.

**Figure 4:**
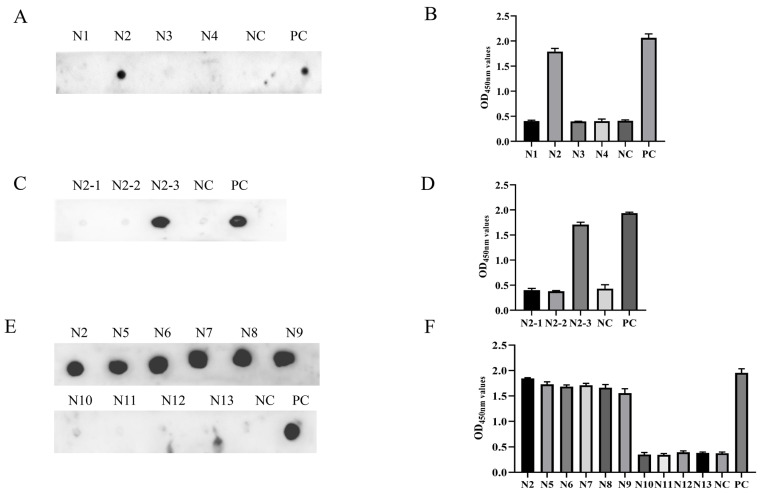
Identification of the antigenic epitope by indirect ELISA and dot blot. (**A**,**B**) MAb 7E3 reactivity with the truncated fragments N1-N4 determined by indirect ELISA and dot blot. (**C**,**D**) MAb 7E3 reactivity with the truncated fragments N2-1, N2-2, and N2-3 determined by indirect ELISA and dot blot. (**E**,**F**) MAb 7E3 reactivity with the truncated fragments N5-N13 determined by indirect ELISA and dot blot. The recombinant GST-Cap protein was employed as positive control (PC), and the empty vector was employed as negative control (NC).

To locate the epitope of the Cap protein, we utilized the IEDB Analysis Resource online software (http://tools.immuneepitope.org/main/, accessed on 28 January 2022) to predict the B cell epitope. This resulted in the division of Cap into a series of truncated and superimposed GST-fused fragments (Figure 3A). Western blotting showed that the fragments were expressed in *E. coli* (Figure 3B). MAb 7E3 had the highest binding affinity for the Cap protein and was chosen for the screening of antigenic epitopes. The Cap protein was constructed into four fragments: N1 (1–60 aa), N2 (61–124 aa), N3 (125–178 aa), and N4 (179–214 aa) (Figure 3A). The results of both the ELISA and dot blot indicated that among the N1–N4 truncated proteins, only the N2 fragments reacted with mAb 7E3 (Figure 4A,B). N2 was then truncated into three fragments: N2–1 (61–95 aa), N2–2 (80–110 aa), and N2–3 (90–124 aa) (Figure 3A), and the results indicated that mAb 7E3 reacted specifically with N2–3 fragments only (Figure 4C,D). This indicated that the targeting epitope was located within 110–124 aa. To further identify the critical region of the epitopes, the fragments were truncated from their N-term and C-termini by two amino acids (Figure 3A). The results showed that N5–N9 could react with mAb 7E3, unlike the N10–N13 truncation (Figure 4E,F). These results demonstrate that 110–116 aa are at the minimum necessary for mAb 7E3 interaction. 

**Figure 5:**
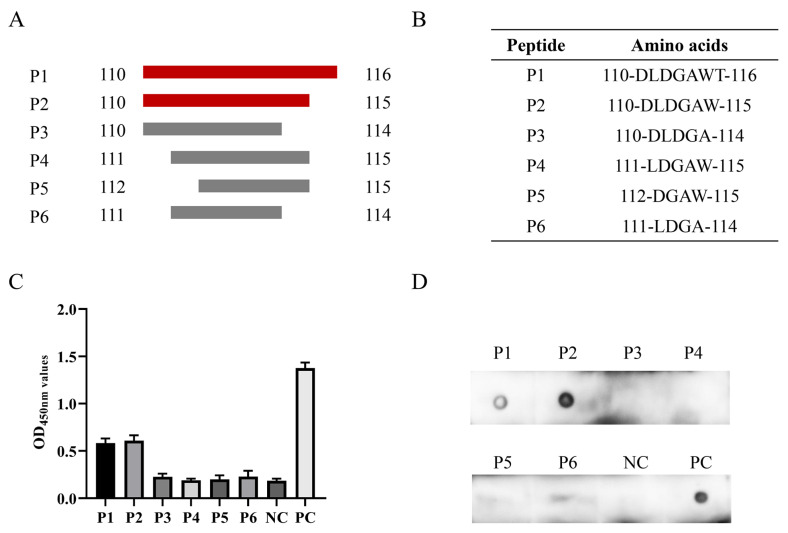
Identification of the minimal 7E3 epitope. (**A**,**B**) The strategies and sequence for synthesizing the peptides. (**C**,**D**) Peptides were detected for the reactivity with mAb 7E3 by indirect ELISA and dot blot assays. The recombinant GST-Cap protein was applied as PC and GST was used as NC.

To determine the minimal B cell epitope, six short peptides were designed and synthesized for subsequent experiments (Figure 5A,B). The ELISA and dot blot results indicated that mAb 7E3 generated a specific reaction with P1 and P2 and did not react with P3–P6 (Figure 5C,D). All data obtained above indicated that ^110^DLDGAW^115^ is the minimal B cell epitope of the PCV3 Cap protein recognized by mAb 7E3.

### 3.4. Establishment of EB-ELISA Based on Cap Protein and mAb 7E3

**Figure 6:**
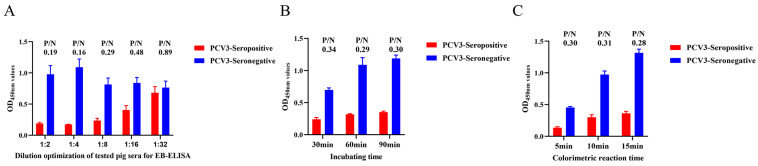
Optimization of developed EB-ELISA reaction conditions. (**A**) Different dilution of the tested porcine sera. OD_450_ value of positive or negative serum samples at different dilutions was measured using EB-ELISA. (**B**) Determine the incubation time after addition of the antibody to be examined**.** (**C**) Optimal time for color development with the addition of TMB substrate.

To verify the diagnostic value of the newly generated mAb 7E3, an EB-ELISA was developed to detect PCV3 antibodies in serum samples. Optimal concentrations of Cap protein and the blocking of mAb-HRP 7E3 were determined using a checkerboard titration assay. The results indicated that the optimum ratio of the two was achieved when the concentration of Cap protein was 16 μg/mL and the dilution level of mAb-HRP 7E3 was 1:400 (Table 1). After testing positive and negative porcine sera in the EB-ELISA, it was determined that the optimal dilution level of porcine serum for testing was 1:4 (Figure 6A). The effective incubation time was found to be 60 min, resulting in the lowest P/N value among the three time points (Figure 6B). Additionally, the colorimetric measurements revealed that the lowest P/N value was achieved after 15 min of incubation (Figure 6C).

Based on the optimized reaction conditions, EB-ELISA for PCV3 antibody detection was performed as follows: 16 μg/mL GST-Cap protein was coated in the 96-well plate at 4 °C overnight after blocking the plates with PBST containing 5% FBS at 37 °C for 1 h. After the wash step, a total of 100 µL of the mixture to be tested, containing 50 µL serum sample for testing (diluted 1:4 with PBST) and 50 µL mAb-HRP 7E3 (diluted 1:400 with PBST), was co-incubated for 1 h at 37 °C. Then, 100 µL TMB substrates was applied for a color reaction at 37 °C for 15 min, and 50 µL 2 mol/L H_2_SO_4_ was applied to stop the reaction. Finally, the plates were read at 450 nm for <10 min using an automatic ELISA microplate reader.

### 3.5. Cut-Off Value for the EB-ELISA

**Figure 7:**
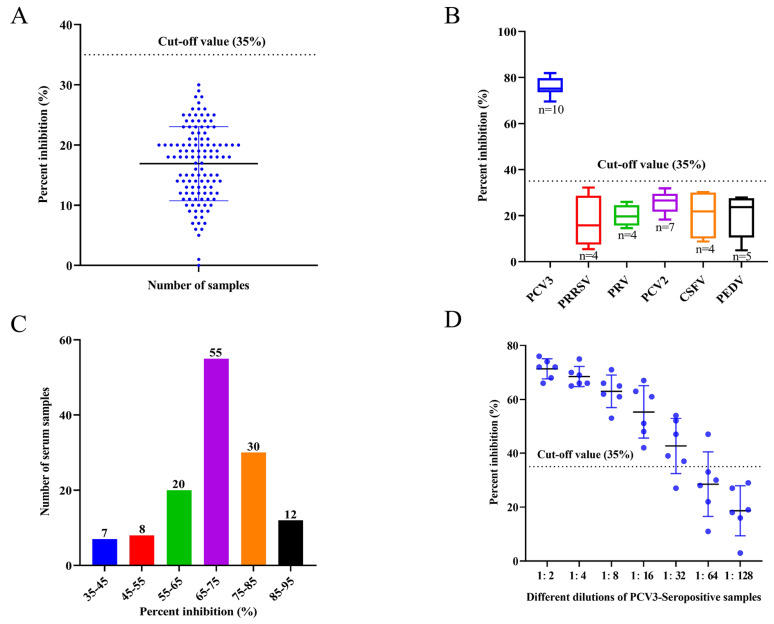
Specificity and sensitivity of the EB-ELISA for detecting anti-PCV3 antibodies. (**A**) In total, 120 PCV3-Seronegative serum samples were tested using EB-ELISA for calculating the cut-off value. (**B**) The blocking rate of porcine virus sera was measured by the developed EB-ELISA to determine the specificity of this assay. PCV3 serum samples tested had PI values above the cut-off value, while the rest of the samples had PI values below the cut-off value. (**C**) Distribution of PI values of clinically identified PCV3-positive sera by EB-ELISA. (**D**) Maximum detection limit of PCV3-positive serum by the developed EB-ELISA.

The cut-off value was determined after a total of 120 negative porcine serum samples were tested using the established EB-ELISA. The results obtained that the mean PI value of the negative serum samples was 17%, and the SD value was determined as 6%. The cut-off value for EB-ELISA was determined to be 35% (17% + 3 SD) (Figure 7A); therefore, it can be judged that when the PI value of the tested serum is ≥35%, the serum is positive. However, all the samples tested negative.

### 3.6. Specificity, Sensitivity, and Repeatability of EB-ELISA

To evaluate the specificity of the developed EB-ELISA, serum samples positive for other porcine disease viruses, including PRRSV, PRV, PCV2, CSFV, and PEDV, were detected by the EB-ELISA. The results indicated that the blocking rate was below the cut-off value, except for PCV3 serum samples (Figure 7B). This indicates that the developed EB-ELISA method has excellent specificity for the detection of clinical PCV3 sera.

To determine the sensitivity of the developed EB-ELISA, 132 porcine serum samples positive for anti-PCV3 antibodies were detected, with PI values ranging from 35% to 91% (Figure 7C). In addition, for the different dilutions of the 6 positive porcine serum samples randomly selected from the above 132 positive samples, 6 samples were detected below the cut-off value at a dilution of 1:128 by the EB-ELISA; after 1:64 dilution, only one sample had a PI value higher than the cut-off value, and one sample was negative when diluted 1:32. Therefore, it may be determined that the maximum dilution of the established EB-ELISA for most positive serum samples was 1:32 (Figure 7D), indicating that the method had good analytical sensitivity.

For the repeatability experiment, three positive and negative random serum samples were detected using the established EB-ELISA, and intra- and inter-batch reproducibility was determined by calculating CV%. In this study, the results showed that the range of intra-assay CVs was 1.47% to 7.59%, and the range of inter-assay CVs was 3.29% to 9.69% (Table 2). The developed EB-ELISA has excellent reproducibility when the CV% was less than 10%.

### 3.7. Agreement of EB-ELISA and Commercial ELISA Kit

To determine the clinical application value of the developed EB-ELISA, 68 clinical porcine serum samples were tested to compare the concordance rate between the EB-ELISA and a commercial ELISA kit. The results indicated that the agreement rate between the two different detection methods was 95.59% (65/68) for porcine serum samples (Table 3). Furthermore, the kappa values show calculations that indicate high compliance with EB-ELISA and commercial ELISA kits (kappa value = 0.75) (Table 3), with the results showing that the established EB-ELISA had good application prospects for detecting the PCV3 antibody.

## 4. Discussion

PCV3 was first discovered in 2016 via metagenomic sequencing in a domestic swine population in the USA [8]. The virus is currently spreading rapidly across the globe and has been identified in multiple countries [29,30,31,32]. Co-infections with PCV3 and PCV2 complicate diagnosis and lead to increased morbidity and mortality [30,33,34]. Owing to the lack of knowledge regarding the protective immunity of PCV3 antigens and an understanding of the diversity of these protective antigens, the progress of PCV3 vaccine development and disease control has been hindered.

The PCV3 Cap protein, a structural viral protein, is necessary for virion packaging and is the main diagnostic target and vaccine candidate [18,19]. The Cap protein is the major antigen recognized by convalescent antisera and is the preferred candidate for serological diagnostics [35]. In this study, four mAbs were prepared after the immunization of mice with prokaryotic recombinant Cap protein. To exclude the influence of the tag antibody on mAb identification, a GST-fused Cap protein was constructed which was different from the His-fused Cap protein of the immune antibody (Figure 1). The results indicated that four mAbs reacted well with the recombinant proteins specifically, with high antibody potencies ranging between 1:2.56 × 10^4^ and 1:1.024 × 10^5^ (Figure 2A). Western blotting and IFA results revealed that the four mAbs could specifically react with GST-fused recombinant Cap protein and recognize Cap protein expressed in 293T cells (Figure 2C,D). These results indicate that the mAbs obtained in the present study were able to specifically recognize the PCV3 Cap protein.

The broad use of B cell epitopes will be of great significance for the diagnosis of pathogens and the design of molecular vaccines. Several antigenic domains of PCV3 Cap have been identified using mouse mAbs. Three linear B cell epitopes, PCV3 Cap ^57^NKPWH^61^, ^140^KHSRYFT^146^, and ^161^QSLFFF^166^, were confirmed as short N- and C-terminal-truncated peptides [36]. It has been demonstrated by PCV2-PCV3 epitope exchange assays that the epitope of mAb-1H11 of PCV3 was located in the CD loop region (72–79 aa) on the VLP surface [22]. To date, only a few linear B cell epitopes of the PCV3 Cap protein have been found and reported, and epitope mapping of the antigenic PCV3 Cap protein has not yet been accomplished. In this study, we prepared mAbs and identified a novel B cell epitope for the PCV3 Cap protein. The four mAbs prepared in the present study belonged to identical subclasses (Figure 2B), and one mAb (7E3), which showed the highest reactivity with the Cap protein in the ELISA and Western blotting, was selected as the antigenic epitope of the Cap protein (Figure 2). The recognized epitope region was determined using a series of truncated GST-fused Cap proteins and screened using an indirect ELISA and dot blotting (Figure 4). To validate a more precise epitope, six overlapping short peptides were artificially synthesized. Therefore, we localized that ^110^DLDGAW^115^ was the shortest B cell epitope of the PCV3 Cap protein recognized by mAb 7E3 (Figure 5). A novel B cell epitope ^110^DLDGAW^115^ was first reported for PCV3 in this study, providing valuable information for studying the structural and functional characteristics of the PCV3 Cap protein.

PCV3 produces antibodies in the infected host for a short period of time and persists for several months [8,37]; thus, an early and accurate diagnosis of PCV3 is valuable. Therefore, it is essential to establish an efficient serologic diagnostic method for the rapid and sensitive laboratory detection of PCV3 infection. IHC and indirect ELISA are the most common diagnostic methods for detecting PCV3 antibodies [8,18,21,25,38]. Antibodies specific to the virus in the serum bind to the pre-coated antigen, and this binding blocks the mAb from binding to the antigen, so the blocking ELISA has good specificity. This method has been widely used for the accurate and specific serological diagnosis of various diseases [39,40,41]. In this study, we innovatively established an EB-ELISA method for PCV3 antibody detection using the obtained mAb. The epitope identified mAb 7E3 as a blocking antibody conjugated to HRP. The method established in this study eliminates the step of incubating the antibody multiple times, which greatly saves time and costs. The cut-off PI value of the EB-ELISA was determined to be 35% by testing 120 SPF-negative porcine serum samples (Figure 7A). The EB-ELISA developed in this study exhibited excellent specificity and no cross-reactivity with sera positive for PRRSV, PRV, PCV2, CSFV, or PEDV (Figure 7B). The developed EB-ELISA provided a highly sensitive detection of antibodies against PCV3 (Figure 7C,D). The intra- and inter-assay comparisons revealed good repeatability (Table 2). The antibodies were detected using the developed EB-ELISA, and 95.59% concordance was achieved compared to the results of the commercial ELISA (Table 3).

## 5. Conclusions

In summary, four hybridoma cell lines producing mAbs against PCV3 were obtained, and the PCV3 Cap protein antigenic epitope ^110^DLDGAW^115^ was identified. Subsequently, using the screened mAb 7E3, an EB-ELISA was successfully developed to detect PCV3 antibodies in porcine sera. This established EB-ELISA has the advantages of being rapid, highly sensitive, reproducible, specific, and did not react with other porcine virus sera, which has great potential for the detection of PCV3 antiserum in porcine farms.

## Figures and Tables

**Table 1:** Identification of the optimal concentration of PCV3-Cap protein for encapsulation and the optimal dilution of PCV3-7E3-HRP mAb.

Coated PCV3-Cap Protein (μg/mL)	OD_450_ Value of Different Dilutions of PCV3-7E3-HRP				
	1:50	1:100	1:200	1:400	1:800
32	2.295	1.868	1.663	1.235	0.967
16	1.815	1.55	1.405	1.025	0.737
8	1.496	1.228	1.117	0.829	0.472
4	0.867	0.623	0.684	0.574	0.331
2	0.445	0.409	0.308	0.290	0.217

**Table 2:** Reproducibility of the EB-ELISA determined by CV% value of intra- and inter-assay.

Type of Precision	CV% Value Range	Median Value
Intra-assay precision (CV%)	1.47–7.59	4.53
Inter-assay precision (CV%)	3.29–9.69	6.49

**Table 3:** Comparisons of the developed EB-ELISA with commercial ELISA kit by detecting clinical porcine serum samples.

Samples	EB-ELISA	Number	Commercial ELISA Kit		Agreement (%)	Kappa Value
			+	−		
Clinical sera	+	60	60	0	95.59	0.75
	−	8	3	5		

## Data Availability

All available data are presented in this manuscript.

## Supplementary Materials

The following supporting information can be downloaded at: https://www.mdpi.com/article/10.3390/ani14020235/s1, Table S1: Primers used for identification of the B cell epitope in PCV3 Cap protein.
