# Supplementary material for: Preparation of Monoclonal Antibodies against the Capsid Protein and Development of an Epitope-Blocking Enzyme-Linked Immunosorbent Assay for Detection of the Antibody against Porcine Circovirus 3
Source: Animals (Basel). 2024 Jan 11;14(2):235. doi: 10.3390/ani14020235 (PMC10812811; doi:10.3390/ani14020235)
Supplement: Supplementary file 1 [file animals-14-00235-s001.zip › animals-2768297-supplementary.pdf]

Table S1. Primers used for identification of the B cell epitope in PCV3 Cap protein.

Primers	Sequences (5’-3’)	Location(aa)
N1	F: CGCGGATCCGCGATGAGACACAGAGCTATATT	1-60
	R: CCGCTCGAGCGGTTACCAGGGCTTGTTATTCT	
N2	F: CGCGGATCCGCGATGCACGCCAACCACTTCAT	61-124
	R: CCGCTCGAGCGGTTAGTCGTCTTGGAGCCAAG	
N3	F: CGCGGATCCGCGATGCCTTATGCGGAAAGTTC	125-178
	R: CCGCTCGAGCGGTTAGGGGTCATATGTGTTGA	
N4	F: CGCGGATCCGCGATGACCGTTCAATGGGGAGC	179-214
	R: CCGCTCGAGCGGTTAGAGAACGGACTTGTAAC	
N2-1	F: CGCGGATCCGCGATGCACGCCAACCACTTCAT	61-95
	R: CCGCTCGAGCGGTTAAGAAATTACAGGGCTGA	
N2-2	F: CGCGGATCCGCGATGTATAAGATACTAAAGAT	80-110
	R: CCGCTCGAGCGGTTAATCTATGGCTGTGTGCC	
N2-3	F: CGCGGATCCGCGATGAGCCCTGTAATTTCTCCAG	90-124
	R: CCGCTCGAGCGGTTAGTCGTCTTGGAGCCAAGT	
N5	F: CGCGGATCCGCGATGCACGCCAACCACTTCAT	61-122
	R: CCGCTCGAGCGGTTATTGGAGCCAAGTGTTT	
N6	F: CGCGGATCCGCGATGCACGCCAACCACTTCAT	61-120
	R: CCGCTCGAGCGGTTACCAAGTGTTTGTGGTCC	
N7	F: CGCGGATCCGCGATGCACGCCAACCACTTCAT	61-118
	R: CCGCTCGAGCGGTTAGTTTGTGGTCCAGGCGC	
N8	F: CGCGGATCCGCGATGCACGCCAACCACTTCAT	61-116
	R: CCGCTCGAGCGGTTAGGTCCAGGCGCCGTCTAG	
N9	F: CGCGGATCCGCGATGGATCTAGACGGCGCCTGGA	110-214
	R: CCGCTCGAGCGGTTATTAGAGAACGGACTTGTAAC	
N10	F: CGCGGATCCGCGATGGACGGCGCCTGGACCACAA	112-214
	R: CCGCTCGAGCGGTTATTAGAGAACGGACTTGTAAC	
N11	F: CGCGGATCCGCGATGGCCTGGACCACAAACACT	114-214
	R: CCGCTCGAGCGGTTATTAGAGAACGGACTTGTAAC	
N12	F: CGCGGATCCGCGATGACCACAAACACTTGGCTC	116-214
	R: CCGCTCGAGCGGTTATTAGAGAACGGACTTGTAAC	
N13	F: CGCGGATCCGCGATGAACACTTGGCTCCAAGACGAC	118-214
	R: CCGCTCGAGCGGTTATTAGAGAACGGACTTGTAAC	
